# Ultrasound elastography in dogs: Physical principles and application in intestinal evaluation

**DOI:** 10.14202/vetworld.2024.2985-2991

**Published:** 2024-12-30

**Authors:** Iago Martins Oliveira, Wanessa Patrícia Rodrigues da Silva, Rafaela Rodrigues Ribeiro, Mariana Moreira Lopes, Paulo Renato dos Santos Costa, Naida Cristina Borges

**Affiliations:** 1 Programa de Pós-graduação em Ciência Animal, Escola de Veterinária e Zootecnia, Universidade Federal de Goiás, Rodovia Goiânia - Nova Veneza, km 8, Campus Samambaia, 74690-900 Goiânia, Goiás, Brasil; 2 Escola de Ciências Médicas e da Vida, Pontifícia Universidade Católica de Goiás, Câmpus II, Av. Engler, s/n - Jardim Mariliza, 74885-460, Goiânia, Goiás, Brasil; 3 Departamento de Veterinária, Universidade Federal de Viçosa, Avenida PH Rolfs, s/n Campus Universitário, 36570.900, Viçosa, Minas Gerais, Brasil; 4 Departamento de Medicina Veterinária, Escola de Veterinária e Zootecnia, Universidade Federal de Goiás, Rodovia Goiânia - Nova Veneza, km 8, Campus Samambaia, 74690-900 Goiânia, Goiás, Brasil

**Keywords:** canine elasticity, intestine elasticity, tissue stiffness

## Abstract

Ultrasound elastography provides diagnostic information based on tissue elasticity. There is a lack of specific studies on the application of elastography in canine intestinal assessment. Therefore, we reviewed comparative medicine studies and those referring to the literature listed in the databases. Static and dynamic elastography techniques are widely applied in human intestinal diseases, especially Chron’s disease, but few studies have investigated the application of these modalities in canine enteropathies. This case raises questions about the use of new diagnostic imaging techniques in veterinary gastroenterology and highlights the need for further research. Hence, this study aimed to review the literature on the physical principles of elastography and its clinical application in the intestinal evaluation of dogs.

## Introduction

Elastography is a non-invasive ultrasound technique that evaluates tissue elasticity, providing insights into structural rigidity [1, 2]. It has emerged as a promising tool for assessing tissue hardness based on elastic models for imaging tissue mechanical properties [3, 4].

The ultrasound technique consists of performing palpation in a technologically advanced manner, yielding more detailed information on shape and rigidity during semiologic evaluation [4]. Previous studies [5, 6] involving intestinal evaluation through elastography described the technique performed on rodents as experimental units following the induction of a Crohn’s disease-analogous model. Animals with intestinal fibrosis had greater tissue stiffness than those with enteritis alone [5, 6]. Similar findings were reported in a study evaluating the jejunum of dogs, but the results were only partially published [7]. The most recent data on this technique in canine intestinal assessment were reported in a case report by Cordella *et al*. [7], which suggested the presence of fibrosis in the lamina propria of the mucosa.

Only a few studies on this subject [5–7] are available in the literature. Considering the potential application of elastography in recognizing fibrosis, staging enteropathies, and aiding prognosis, it is important that there are publications on the subject. Therefore, we conducted a literature review on the general principles of elastography and its application in the intestinal evaluation of dogs.

## Ultrasound Elastography

### General principles of elastography

Elastography in medicine was first introduced by Ophir *et al*. [4]; after that, it was found that the test can assess tissue mobility during compression and amplify displacement along the axis of movement, with displacement varying according to tissue rigidity parameters. Elasticity, the property enabling a structure to deform under applied force and revert to its original shape and size after the force has been removed, is inversely proportional to the stiffness and recovery period of the tissue [8]. The generated deformity is then converted into elastic moduli to form images [4].

Elasticity is influenced by the molecular constitution and morphological organization of microscopy and macroscopy, with factors such as fat, collagen, elastin, and water content affecting organ elasticity [9]. Soft tissue biomechanics depend on the molecular composition of the tissue and affect its elasticity, rigidity, and mobility under an applied force [10].

**Figure-1:**
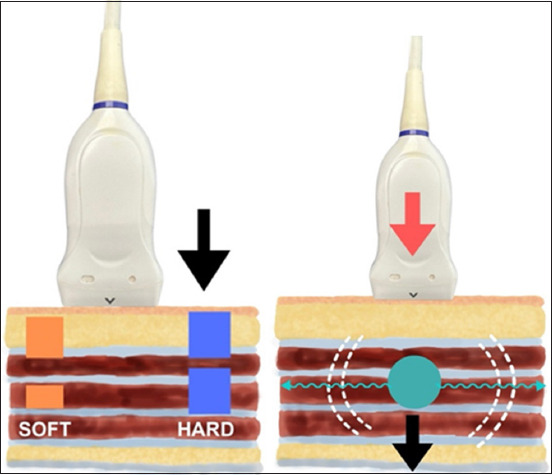
Schematic representation of elastography modalities. (a) Elastography with compression or deformation, which measures the displacement of the tissue along the axis where the force is exerted. (b) Elastography with shear waves generated by the tangential sliding of the tissue particles. The figure was created using the Penup Samsung® application (Windows 11, Ldta, Samsung Electronics, Suwon, South Korea) and Canva Pro® (Canva Pty Ltd, Sydney, Australia).

Elastography can be classified into static and dynamic [11] (Figure-1). Over the years, several elastography modalities have been developed and proposed. Static techniques involve compression, tension, or deformation, whereas dynamic methods include sonoelastography, elastography with acoustic radiation force impulse (ARFI), two-dimensional shear-wave elastography (SWE-2D), and supersonic shear-wave imaging [12–14]. Table-1 lists a compilation of studies on the types of elastography and the authors [3, 15–17].

**Table-1:** Elastography modalities and characterization results in terms of applied force, vibration source, and advantages and disadvantages.

Technique	Force applied	Vibration source	Advantages	Disadvantages	References
Elastography with compression	Mechanical compression	Manual or automatic	Accessible	Dependent operator Sensitive to body conditions such as abdominal effusion and obesity	[3, 15–17]
Sonoelastography	Automatic compression	The transient mechanical force	Medical validation; Use in liver fibrosis	Onerous Longer learning curve Sensitive to body conditions such as abdominal effusion and obesity	[3, 15–17]
supersonic Image	Shear wave	Acoustic radiation force impulse	Faster Independent operator	Onerous Little standardization Few studies	[3, 15–17]
Elastography ARFI	Shear wave	Acoustic radiation force impulse	Application to all bodies Independent operator	Onerous Little standardization Few studies	[3, 15–17]

ARFI=Acoustic radiation force impulse

Freehand elastography is also known as static, compression, or deformation elastography [18]. It measures the time interval between radiofrequency echo signals before and after compression, either by the examiner or by the internal physiological movements of the organs [1, 19]. In this method, the tissue under assessment is compressed by applying a force, following which the imaging program measures the tissue’s response to the force applied by mechanical or automatic compression [20, 21]. The resulting image was generated by quantifying the deformation induced in the structure. A specific software facilitates the comparative analysis between the compression moment and its generated result [21].

**Figure-2:**
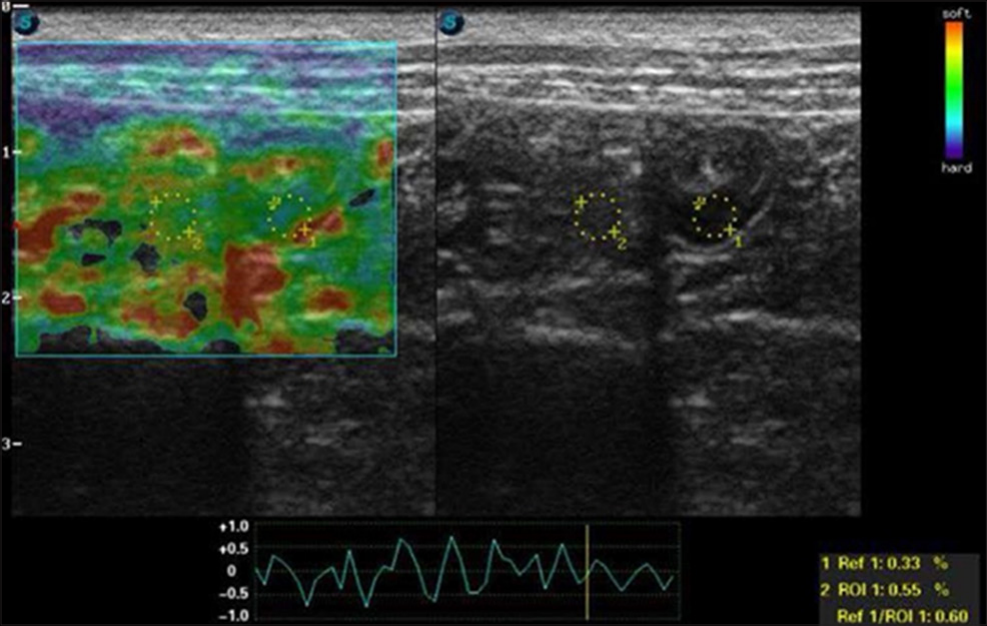
Cross-sectional B-mode ultrasound image (left) of the duodenum of a healthy dog, showing preserved stratification of layers and thickness, echogenicity, and echotexture under normal ultrasound parameters. The corresponding color elastogram obtained by compression elastography on a SAEVO® FT422 device (Alliage S/A, Ribeirão Preto, São Paulo, Barazil) (right) shows a similar stiffness profile to that of the surrounding normal mesentery (region of interest <1), suggesting normality.

To perform this examination, several compressions are required with the transducer positioned over the study area, accompanied by decompression cycles. The pressure exerted should be gentle, compressing from 2 to 5 mm [1, 3]. The deformation ratio can be determined by calculating the stress in the normal region of interest (ROI) and the ROI of the altered structure. A deformation ratio >1 indicates that the injured area deforms less compared to adjacent healthy tissue, indicating greater tension or hardness [22] (Figure-2).

**Figure-3:**
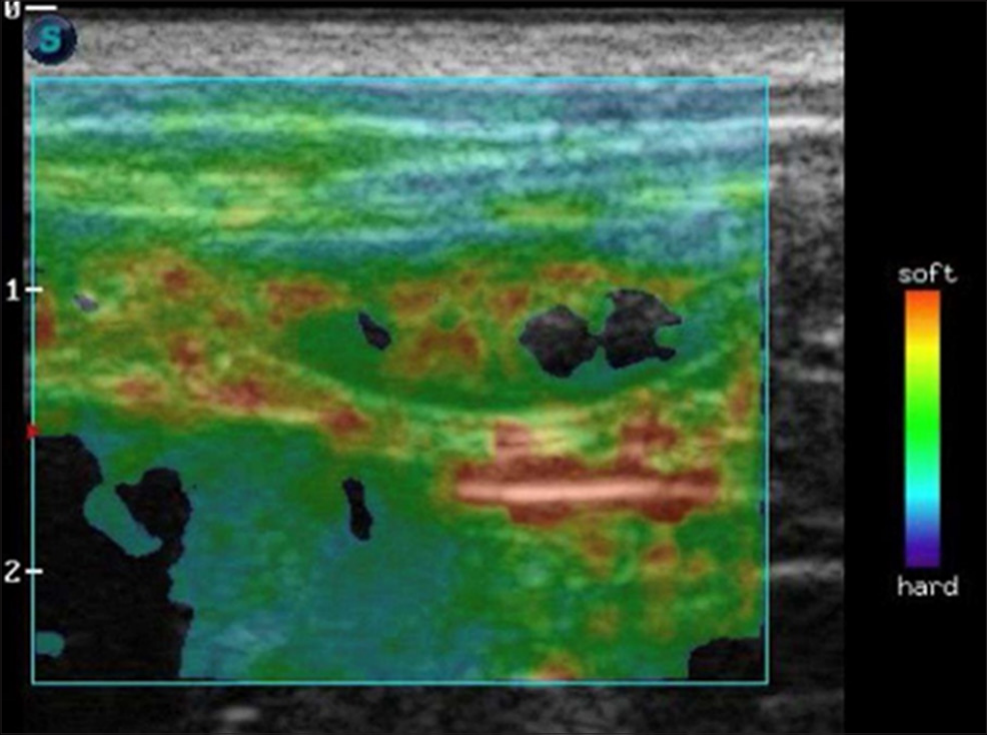
Image of an elastogram of the intestinal loop of a dog’s duodenum in the longitudinal section and adjacent mesentery, showing the scale of interpretation of the color nuances of the elastogram between the red (soft) and blue (hard) color extremes.

Firmer materials deform less under an applied force, whereas softer materials deform more. This deformation is represented on a colorimetric scale or color map overlaying the B-mode image according to elastic variation, with red corresponding to softer tissues, green to intermediate deformity, and blue tissues to greater hardness, i.e., those with less deformation [1, 3, 21] (Figure-3). However, the color scale varies depending on the ultrasound device. Tissues with low tension or increased hardness exhibit lighter-tone images, whereas those with increased tension and low hardness display darker colors [15, 23].

To perform strain elastography, it is necessary to trichotomize the evaluation region. The evaluator should position the transducer perpendicularly without lateralizing the probe and initiate the examination with proper transducer positioning, followed by compression and decompression cycles [3, 24]. Although this technique is validated with greater accessibility, it has the disadvantage of being influenced by the patient’s body shape and deeper organs and being overweight. In addition, it depends on the operator’s pressure and force exerted during the assessment [16].

In dynamic techniques, shear waves and sonoelastography generate images based on movement waves. In these experiments, a force is applied to the tissue, and the imaging program measures the generated response. Qualitative elastography converts the calculations derived from the applied force into images by inducing longitudinal or axial displacement across different regions of the same tissue. This process generates an elastogram [20].

In sonoelastography, mechanical vibrations of low amplitude and frequency are transmitted to the tissue after selecting the image for evaluation. This generates an elastic shear wave that propagates, providing elements for qualitative evaluation through elastographic images. Quantitative data are determined kilopascal (kPa) [16, 25]. It is considered a more modern imaging test that yields objective data, and the numerical hardness information is determined by a color scale based on elasticity and expressed in kPa [26].

In SWE-2D, the radiation diverges from the tissue in different directions and evaluates focal regions (ROIs), creating a shear mark resembling a cylindrical geometric figure. This enables dynamic wave monitoring in 2D. In these cases, the elastogram combined qualitative data from the colorimetric map with quantitative information in m/s or kPa. These metrics can be used to differentiate structures during an ultrasound with elastography [14].

ARFI employs an acoustic wave directed toward the target structure. This process determines the propagation of pressure impulses that deform the tissue, subsequently obtaining the shear velocity in m/s, which characterizes the qualitative aspect of the technique [3]. These pulses, while short, have high intensity and can be conducted in conjunction with the B-mode technique or with the image projected onto the side of the screen to optimize the comparison of the generated color tones. In this method, variability between examiners is reduced because there is no need for manual compression [27].

Thus, ARFI elastography yields qualitative results using acoustic pulses of the above-mentioned duration and speed to deform tissues, forming the elastogram on gray scales similar to the images formed by B-mode ultrasound. The grayscale serves as a tool to indicate the relative rigidity of the explored tissue, with lighter areas indicating more deformable or less rigid materials [8].

Several studies [28–36] have investigated the application of elastography to different organs in veterinary medicine due to its beneficial characteristics. This is particularly important because it is a non-invasive and safe technique that does not require sedation of the animals and can be combined with other diagnostic imaging techniques. It is also a potentially effective alternative to invasive diagnostic methods [28]. Recent studies have demonstrated the use of elastography for the assessment of various organs, such as the prostate [29], liver [30], kidneys [31], spleen [32], lymph nodes [33], adrenal glands [34], thyroid [35], hip joint, and mammary neoplasms [36].

### Intestinal elastography

Intestinal elastography is widely used in medicine. Some studies use this technique in the diagnosis of intestinal stenosis before the surgical procedure of intestinal resection [37]; a systematic review on the correlation between elastography and histopathological aspects in inflammatory bowel disease, the aim of which was to verify whether the increased hardness of the intestinal mucosa was related to histological structural changes [38]; research into the application of this examination in distinguishing between inflammatory and fibrotic changes [39], a specific review of elastography in Crohn’s disease [40] and a review of the compression technique based on visual assessment and semi-quantitative intestinal parameters [41].

The physiological elasticity of the jejunal mucosa of healthy dogs was assessed using shear wave elastography, as there is not much data on this technique for the gastrointestinal tract compared with other abdominal organs. The results determined normal ranges (1305–9319 kPa) for jejunal mucosal stiffness in healthy dogs. This technique was effective and safe and did not require sedation or anesthesia. These preliminary findings lay the groundwork for further studies into intestinal mucosal rigidity in enteropathies of various origins [11]. In this study [11], an ultrasound examination was conducted using a Supersonic Imagine Aixplorer scanner and an SL15-4 linear probe (Supersonic Imagine, Aix-en-Provence, France) with a frequency range of 4–15 MHz. Elastography was performed 3 consecutive times by the same evaluator to locate the proximal jejunum.

In a case report involving a French bulldog with granulomatous colitis, ultrasound contrast and elastography were used to assess the colon in conjunction with conventional tests [7]. In the elastographic evaluation of the report by Cordella *et al*. [7], the authors referenced techniques applied to the jejunum of dogs [11] and Crohn’s disease in children [17], highlighting the absence of a defined normal pattern for colon examination in dogs [7]. In this study, when evaluating pediatric cases of Crohn’s disease, the elastogram revealed a normal intestinal wall in the control group, which was characterized by colored stratification. In contrast, diseased individuals exhibit increased muscle rigidity with a greater tendency toward single coloration [17].

In a case report by Cordella *et al*. [7], a thin linear area of greater rigidity was observed in the middle of the colon wall, which was thickened and colored yellow by the software, while the remaining area was homogeneously colored blue, indicating softer tissue. The hypothesis that the firmer linear region observed by elastography could be the lamina propria of the mucosa with fibrosis was suggested, but owing to the lack of reference values, the authors concluded that interpretation should be performed with caution. This technique has shown promise, but more studies on intestinal elastography in dogs are needed.

A recent review of chronic enteropathy in dogs showed that all segments of the small intestine are affected by intestinal inflammation, but there is no difference in prevalence [42]. In humans, the ileum is more affected by inflammatory bowel disease than the other segments, which is why this region is often studied using elastography [43]. We believe that veterinary medicine assessment of the ileum is more difficult than other intestinal segments due to its luminal diameter, mucosal thickness, and presence of gas, which would probably make it difficult to draw ROIs and apply the color histogram.

A medical gastroenterology publication obtained reference measurements using strain elastography, in which RD was 1.5 in moderate intestinal fibrosis and 2.4 in severe fibrosis, with a 95% confidence interval ranging from 0.788 to 1 [44]. There are no reference values for intestinal elastography in dogs that use the deformation technique.

In the qualitative assessment of deformation elastography, standards and scores for dogs are not defined. Unlike human medicine, there is a suggestion of colorimetric standardization by means of scales with scores in which they verified evidence of inflammation and fibrosis of the intestinal wall by means of the color map in three types: Intestine in remission (blue/green/blue), inflamed mucosa (green/blue), and fibrotic mucosa (blue without color stratification). However, this scale has limited clinical applications because it has not been correlated with histological criteria [17].

Some studies by Gabbiadini *et al*. [39] and Salavati *et al*. [45] have evaluated the potential of intestinal elastography for the early detection of fibrosis and stenosis in individuals with chronic enteritis. However, we believe that research in this direction is lacking in small animal medicine because chronic inflammatory enteropathy, which is responsive to immunosuppressants, does not usually evolve into a narrowing of the lumen. To our knowledge, no studies have correlated intestinal elastography with the histological characteristics of the mucosa.

B-mode ultrasound scans of the intestines of dogs show increased echogenicity of the mucosa, wall thickening, loss of the layering pattern, and hyperechogenic striations in the mucosa. Among these alterations, increased intestinal mucosal thickening in patients with inflammatory diseases was also associated with alterations in strain elastography in people. It was found that higher strain rates obtained from semi-quantitative parameters occurred in the thicker intestinal segments observed on ultrasound examination. There are no directly comparable studies with dogs [41].

Based on research involving people with chronic kidney disease, elastography has shown promise in diagnosing increases in kidney tissue stiffness. Given these findings, a study was carried out on cats with chronic kidney disease, and elastography was not an effective tool for diagnosing feline kidney disease [31]. Comparatively and in contrast to the authors, elastography may be promising for detecting fibrosis and helping to stage the inflammatory disease, as well as serving as a prognostic factor since intestinal fibrosis may be related to an unfavorable response to clinical treatment.

Shear-wave elastography effectively predicts malignancy in dogs with splenic lesions [32]. This finding has also been verified in cases of canine mammary neoplasia [28]. However, for differentiating intestinal neoplasms from inflammatory diseases such as chronic immunosuppressant-responsive enteropathy, elastography would probably be restricted to detecting increased mucosal rigidity, given the similarity of the histological lesions seen between alimentary lymphoma, one of the main intestinal neoplasms in dogs and cats, and inflammatory conditions.

A recent study on the elastographic aspects of the adrenal glands of healthy dogs of different ages showed that the stiffness pattern can vary in healthy dogs of different ages, with variations in the poles of the glands [34]. Comparative studies with dogs of different ages and body conditions are needed to establish references for intestinal elastography. Currently only one study exists for the jejunum and shear technique [11]. Studies using deformation elastography and evaluation of other intestinal segments are needed.

The images used in this review were derived from a study on elastography of semiquantitative deformation of the duodenum in healthy dogs and those with chronic inflammatory enteropathy (unpublished data). Because there is no information on standardized techniques in the veterinary literature, preliminary results suggest that the duodenal mucosa of healthy dogs can be evaluated through deformation elastography by examining the ROI within the intestinal mucosa compared with the adjacent mesentery. The technique was found to be easier when a cross-section was made in the proximal segment of the duodenum, accompanied by B-mode ultrasound over a frequency range of 7.5–10 MHz. It was not possible to perform the deformation ratio using the ROI on the abdominal muscle wall because there was no stabilization of the elastographic wave, resulting in homogeneous filling of the mucosa by the elastogram and the inability to longitudinally parallel the ROI of the duodenal mucosa with the abdominal wall.

## Conclusion

In conclusion, ultrasound elastography presents promising potential as a diagnostic tool for assessing intestinal diseases in dogs, drawing from its established success in human medicine, particularly in conditions like Crohn’s disease. Despite its proven efficacy in evaluating tissue elasticity, the application of both static and dynamic elastography in veterinary gastroenterology remains underexplored. Future studies should focus on developing standardized methodologies, validating its clinical relevance in canine enteropathies, and establishing comparative reference values. Advancing this research will bridge the existing knowledge gaps, improve diagnostic precision, and pave the way for wider clinical adoption of elastography in veterinary medicine.

## Authors’ Contributions

IMO and NCB: Drafted and revised the manuscript. WPRS, RRR, and MML: Reviewed the literature and revised the manuscript. WPRS and RRR: Selected the images for this study on strain elastography and synthesized schematic and representative drawings of the topic. PRSC: Drafted the manuscript. All authors have read and approved the final manuscript.
